# 4-Methyl­benzyl­ammonium nitrate

**DOI:** 10.1107/S1600536813022836

**Published:** 2013-08-21

**Authors:** Sofian Gatfaoui, Houda Marouani, Mohamed Rzaigui

**Affiliations:** a Laboratoire de Chimie des Matériaux, Faculté des Sciences de Bizerte, 7021 Zarzouna Bizerte, Tunisia

## Abstract

In the title salt, C_8_H_12_N^+^·NO_3_^−^, the N atom of the 4-methyl­benzyl­ammonium cation is displaced by 1.366 (2) Å from the mean plane of the other atoms. In the crystal, the cations are connected to the anions by N—H⋯O and N—H⋯(O,O) hydrogen bonds, generating a layered network parallel to (100). A weak C—H⋯O inter­action also occurs.

## Related literature

For related structures, see: Kefi *et al.* (2011 ▶); Rahmouni *et al.* (2011 ▶). For a discussion on hydrogen bonding, see: Brown (1976 ▶); Blessing (1986 ▶). For aromatic π–π stacking inter­actions, see: Janiak (2000 ▶). For graph-set notation of hydrogen-bonding patterns, see: Bernstein *et al.* (1995 ▶).
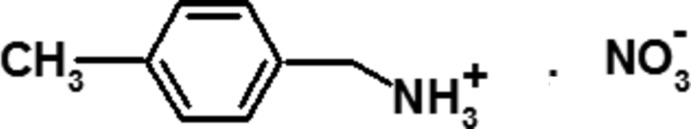


## Experimental

### 

#### Crystal data

C_8_H_12_N^+^·NO_3_^−^
*M* *_r_* = 184.20
Monoclinic, 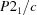

*a* = 15.097 (2) Å
*b* = 5.8121 (10) Å
*c* = 10.486 (2) Å
β = 99.75 (2)°
*V* = 906.8 (3) Å^3^
*Z* = 4
Ag *K*α radiation
λ = 0.56083 Å
μ = 0.06 mm^−1^
*T* = 293 K
0.40 × 0.35 × 0.30 mm

#### Data collection

Enraf–Nonius CAD-4 diffractometer
6579 measured reflections
4430 independent reflections
2415 reflections with *I* > 2σ(*I*)
*R*_int_ = 0.033
2 standard reflections every 120 min intensity decay: 1%

#### Refinement

*R*[*F* ^2^ > 2σ(*F* ^2^)] = 0.071
*wR*(*F* ^2^) = 0.216
*S* = 0.96
4430 reflections
122 parameters
H-atom parameters constrained
Δρ_max_ = 0.31 e Å^−3^
Δρ_min_ = −0.17 e Å^−3^

### 

Data collection: *CAD-4 EXPRESS* (Enraf–Nonius, 1994 ▶); cell refinement: *CAD-4 EXPRESS*; data reduction: *XCAD4* (Harms & Wocadlo, 1995 ▶); program(s) used to solve structure: *SHELXS97* (Sheldrick, 2008 ▶); program(s) used to refine structure: *SHELXL97* (Sheldrick, 2008 ▶); molecular graphics: *ORTEP-3 for Windows* (Farrugia, 2012 ▶) and *DIAMOND* (Brandenburg & Putz, 2005 ▶); software used to prepare material for publication: *WinGX* (Farrugia, 2012 ▶).

## Supplementary Material

Crystal structure: contains datablock(s) I. DOI: 10.1107/S1600536813022836/hb7131sup1.cif

Structure factors: contains datablock(s) I. DOI: 10.1107/S1600536813022836/hb7131Isup2.hkl

Supplementary material file. DOI: 10.1107/S1600536813022836/hb7131Isup3.cml

Additional supplementary materials:  crystallographic information; 3D view; checkCIF report

## Figures and Tables

**Table 1:** Hydrogen-bond geometry (Å, °)

*D*—H⋯*A*	*D*—H	H⋯*A*	*D*⋯*A*	*D*—H⋯*A*
N1—H1*A*⋯O1^i^	0.89	2.07	2.936 (3)	164
N1—H1*A*⋯O3^i^	0.89	2.52	3.065 (2)	120
N1—H1*B*⋯O3^ii^	0.89	2.12	2.9378 (19)	153
N1—H1*C*⋯O3	0.89	2.01	2.900 (2)	179
N1—H1*C*⋯O2	0.89	2.55	3.158 (3)	126
C8—H8*A*⋯O1^iii^	0.97	2.45	3.234 (2)	138

Symmetry codes: (i) 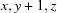; (ii) 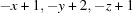; (iii) 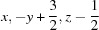.

**Fig. 1.**
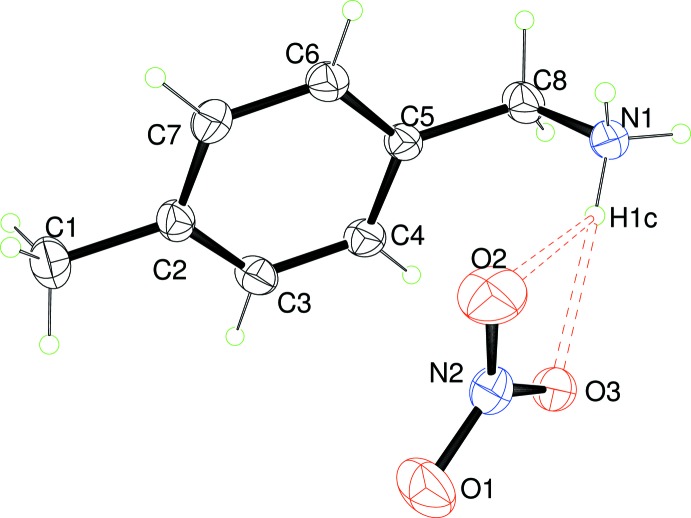
An ORTEP view of (I) with displacement ellipsoids drawn at the 30% probability level. H atoms are represented as small spheres of arbitrary radii. Hydrogen bonds are shown as dotted lines.

**Fig. 2.**
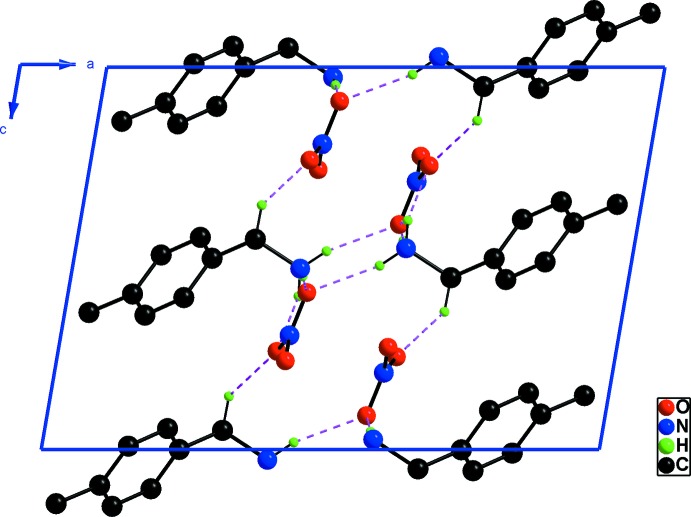
Projection of (I) along the b axis. The H-atoms not involved in H-bonding are omitted.

**Fig. 3.**
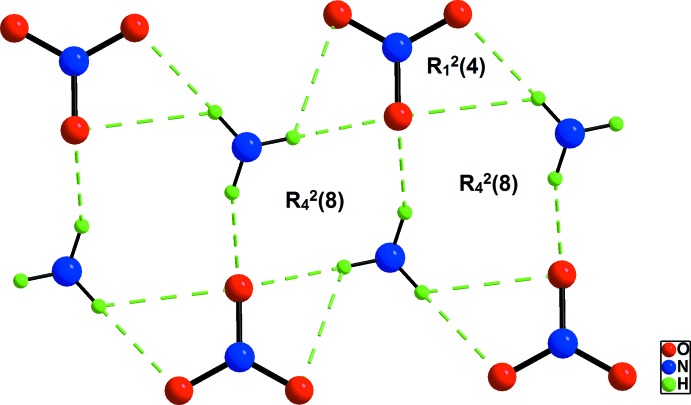
Hydrogen bond motifs in (I).

## supplementary crystallographic information

### 1. Comment

We report here the preparation and the crystal structure of the title compound, C_8_H_12_N·NO_3_ (I).

The asymmetric unit of (I) consists of one nitrate anion and one 4-methylbenzylammonium cation (Figure 1). The 4-methylbenzylammonium cations are connected to the nitrate anions through weak N—H···O and C—H···O hydrogen bonds with donor-acceptor distances varying between 2.900 (2) and 3.234 (2) Å [d (N(C)···O) > 2.73 Å] (Brown, 1976); (Blessing, 1986) (Table 1, Figure 2).

In the nitrate anion, the distance N2—O2 is significantly shorter than the N2—O1 and N2—O3 distances because O2 is applied in only one hydrogen bond (table1) while O1 and O3 are applied in two and three hydrogen bonds, respectively. These geometrical features have also been noticed in other crystal structures (Rahmouni, *et al.*, 2011).

Each organic entity is bounded to three different nitrate anions through five N—H···O hydrogen bonds forming *R*_1_^2^(4) and *R*_4_^2^(8) motifs (Fig. 3) (Bernstein, *et al.*, 1995). Examination of the 4-methylbenzylammonium cation shows that the bond distances and angles show no significant difference from those obtained in other structures involving the same organic groups (Kefi, *et al.*, 2011). The aromatic ring of the organic cation is essentially planar with an r.m.s deviation of 0.0099 Å. The inter-planar distance between nearby phenyl rings is in the vicinity of 5.925 Å, which is much longer than 3.80 Å, value required for the formation of π–π interactions (Janiak, 2000).

The crystal cohesion and stability are ensured by electrostatic and van der Waals interactions which, together with N—H···O and C—H···O hydrogen bonds, build up a two-dimensional network.

### 2. Experimental

An aqueous solution containing 1 mmol of HNO_3_ in 10 ml of water, was added to 1 mmol of 4-xylylamine in 10 ml of ethanol. The obtained solution was stirred for 20 min and then left to stand at room temperature. Colorless prisms of the title compound were obtained after some days.

### 3. Refinement

All H atoms were fixed geometrically and treated as riding with C—H = 0.93 Å (aromatic) or 0.97 Å (methylene) or 0.96 Å (methyl), N—H = 0.89 Å with *U*_iso_(H) = 1.2Ueq(C or N).

### Crystal data

**Table:**

C_8_H_12_N^+^·NO_3_^−^	*F*(000) = 392
*M**_r_* = 184.20	*D*_x_ = 1.349 Mg m^−^^3^
Monoclinic, *P*2_1_/*c*	Ag *K*α radiation, λ = 0.56083 Å
Hall symbol: -P 2ybc	Cell parameters from 25 reflections
*a* = 15.097 (2) Å	θ = 9–11°
*b* = 5.8121 (10) Å	µ = 0.06 mm^−^^1^
*c* = 10.486 (2) Å	*T* = 293 K
β = 99.75 (2)°	Prism, colorless
*V* = 906.8 (3) Å^3^	0.40 × 0.35 × 0.30 mm
*Z* = 4	

### Data collection

**Table:**

Enraf–Nonius CAD-4 diffractometer	*R*_int_ = 0.033
Radiation source: fine-focus sealed tube	θ_max_ = 28.0°, θ_min_ = 2.2°
Graphite monochromator	*h* = −2→25
non–profiled ω scans	*k* = −9→2
6579 measured reflections	*l* = −17→17
4430 independent reflections	2 standard reflections every 120 min
2415 reflections with *I* > 2σ(*I*)	intensity decay: 1%

### Refinement

**Table:**

Refinement on *F*^2^	Primary atom site location: structure-invariant direct methods
Least-squares matrix: full	Secondary atom site location: difference Fourier map
*R*[*F*^2^ > 2σ(*F*^2^)] = 0.071	Hydrogen site location: inferred from neighbouring sites
*wR*(*F*^2^) = 0.216	H-atom parameters constrained
*S* = 0.96	*w* = 1/[σ^2^(*F*_o_^2^) + (0.0879*P*)^2^] where *P* = (*F*_o_^2^ + 2*F*_c_^2^)/3
4430 reflections	(Δ/σ)_max_ = 0.011
122 parameters	Δρ_max_ = 0.31 e Å^−^^3^
0 restraints	Δρ_min_ = −0.17 e Å^−^^3^

### Special details

**Table:**

Geometry. All e.s.d.'s (except the e.s.d. in the dihedral angle between two l.s. planes) are estimated using the full covariance matrix. The cell e.s.d.'s are taken into account individually in the estimation of e.s.d.'s in distances, angles and torsion angles; correlations between e.s.d.'s in cell parameters are only used when they are defined by crystal symmetry. An approximate (isotropic) treatment of cell e.s.d.'s is used for estimating e.s.d.'s involving l.s. planes.
Refinement. Refinement of *F*^2^ against ALL reflections. The weighted *R*-factor *wR* and goodness of fit *S* are based on *F*^2^, conventional *R*-factors *R* are based on *F*, with *F* set to zero for negative *F*^2^. The threshold expression of *F*^2^ > σ(*F*^2^) is used only for calculating *R*-factors(gt) *etc*. and is not relevant to the choice of reflections for refinement. *R*-factors based on *F*^2^ are statistically about twice as large as those based on *F*, and *R*- factors based on ALL data will be even larger.

### Fractional atomic coordinates and isotropic or equivalent isotropic displacement parameters (Å^2^)

**Table:**

	*x*	*y*	*z*	*U*_iso_*/*U*_eq_	
O3	0.42764 (9)	0.7571 (2)	0.58898 (11)	0.0491 (4)	
N2	0.40982 (10)	0.7432 (3)	0.70119 (15)	0.0495 (4)	
N1	0.40818 (11)	1.2425 (3)	0.52659 (18)	0.0576 (5)	
H1A	0.4136	1.3250	0.5990	0.086*	
H1B	0.4500	1.2859	0.4811	0.086*	
H1C	0.4151	1.0940	0.5464	0.086*	
C6	0.20891 (13)	1.2197 (3)	0.60241 (18)	0.0485 (5)	
H6	0.2267	1.3600	0.6411	0.058*	
C2	0.11570 (12)	0.8824 (3)	0.59300 (17)	0.0455 (5)	
C7	0.14446 (13)	1.0904 (4)	0.64823 (18)	0.0510 (5)	
H7	0.1198	1.1447	0.7180	0.061*	
C5	0.24736 (12)	1.1432 (3)	0.49952 (16)	0.0420 (4)	
C4	0.21981 (13)	0.9329 (3)	0.44485 (18)	0.0494 (5)	
H4	0.2453	0.8768	0.3763	0.059*	
C8	0.31895 (14)	1.2799 (4)	0.44969 (19)	0.0538 (5)	
H8A	0.3200	1.2369	0.3606	0.065*	
H8B	0.3042	1.4423	0.4511	0.065*	
C3	0.15502 (13)	0.8059 (3)	0.49093 (19)	0.0517 (5)	
H3	0.1373	0.6654	0.4525	0.062*	
O1	0.39214 (12)	0.5519 (3)	0.74280 (15)	0.0818 (6)	
O2	0.41003 (13)	0.9155 (3)	0.76752 (16)	0.0859 (6)	
C1	0.04360 (14)	0.7443 (4)	0.6414 (2)	0.0632 (6)	
H1D	0.0477	0.7676	0.7329	0.095*	
H1E	0.0516	0.5840	0.6244	0.095*	
H1F	−0.0144	0.7933	0.5977	0.095*	

### Atomic displacement parameters (Å^2^)

**Table:**

	*U*^11^	*U*^22^	*U*^33^	*U*^12^	*U*^13^	*U*^23^
O3	0.0556 (8)	0.0473 (8)	0.0478 (7)	0.0014 (6)	0.0184 (6)	0.0008 (6)
N2	0.0483 (9)	0.0536 (10)	0.0499 (9)	0.0024 (8)	0.0175 (7)	−0.0024 (8)
N1	0.0558 (10)	0.0390 (9)	0.0870 (12)	−0.0007 (8)	0.0379 (9)	0.0063 (9)
C6	0.0555 (11)	0.0418 (10)	0.0504 (10)	−0.0018 (9)	0.0157 (9)	−0.0056 (9)
C2	0.0400 (9)	0.0471 (11)	0.0494 (10)	0.0003 (9)	0.0073 (8)	0.0096 (9)
C7	0.0523 (11)	0.0537 (12)	0.0506 (10)	0.0038 (10)	0.0197 (9)	−0.0026 (9)
C5	0.0471 (10)	0.0377 (9)	0.0428 (9)	−0.0005 (9)	0.0117 (8)	0.0029 (8)
C4	0.0628 (12)	0.0432 (11)	0.0459 (10)	0.0024 (10)	0.0195 (9)	−0.0029 (8)
C8	0.0662 (13)	0.0471 (12)	0.0531 (10)	−0.0053 (10)	0.0245 (10)	0.0021 (9)
C3	0.0607 (12)	0.0409 (11)	0.0541 (11)	−0.0055 (9)	0.0116 (10)	−0.0006 (9)
O1	0.1100 (14)	0.0670 (11)	0.0772 (11)	−0.0136 (10)	0.0413 (10)	0.0139 (9)
O2	0.1169 (15)	0.0737 (12)	0.0739 (10)	0.0027 (11)	0.0353 (10)	−0.0280 (9)
C1	0.0542 (12)	0.0675 (15)	0.0705 (13)	−0.0100 (11)	0.0178 (11)	0.0083 (12)

### Geometric parameters (Å, º)

**Table:**

O3—N2	1.2533 (19)	C2—C1	1.508 (3)
N2—O2	1.219 (2)	C7—H7	0.9300
N2—O1	1.240 (2)	C5—C4	1.384 (3)
N1—C8	1.464 (3)	C5—C8	1.505 (2)
N1—H1A	0.8900	C4—C3	1.376 (3)
N1—H1B	0.8900	C4—H4	0.9300
N1—H1C	0.8900	C8—H8A	0.9700
C6—C7	1.379 (3)	C8—H8B	0.9700
C6—C5	1.383 (2)	C3—H3	0.9300
C6—H6	0.9300	C1—H1D	0.9600
C2—C7	1.378 (3)	C1—H1E	0.9600
C2—C3	1.382 (3)	C1—H1F	0.9600
			
O2—N2—O1	121.05 (17)	C4—C5—C8	120.39 (16)
O2—N2—O3	120.18 (18)	C3—C4—C5	120.63 (17)
O1—N2—O3	118.77 (17)	C3—C4—H4	119.7
C8—N1—H1A	109.5	C5—C4—H4	119.7
C8—N1—H1B	109.5	N1—C8—C5	112.22 (16)
H1A—N1—H1B	109.5	N1—C8—H8A	109.2
C8—N1—H1C	109.5	C5—C8—H8A	109.2
H1A—N1—H1C	109.5	N1—C8—H8B	109.2
H1B—N1—H1C	109.5	C5—C8—H8B	109.2
C7—C6—C5	120.72 (18)	H8A—C8—H8B	107.9
C7—C6—H6	119.6	C4—C3—C2	121.56 (19)
C5—C6—H6	119.6	C4—C3—H3	119.2
C7—C2—C3	117.47 (17)	C2—C3—H3	119.2
C7—C2—C1	121.30 (18)	C2—C1—H1D	109.5
C3—C2—C1	121.2 (2)	C2—C1—H1E	109.5
C2—C7—C6	121.50 (18)	H1D—C1—H1E	109.5
C2—C7—H7	119.3	C2—C1—H1F	109.5
C6—C7—H7	119.3	H1D—C1—H1F	109.5
C6—C5—C4	118.11 (17)	H1E—C1—H1F	109.5
C6—C5—C8	121.48 (17)		

### Hydrogen-bond geometry (Å, º)

**Table:**

*D*—H···*A*	*D*—H	H···*A*	*D*···*A*	*D*—H···*A*
N1—H1*A*···O1^i^	0.89	2.07	2.936 (3)	164
N1—H1*A*···O3^i^	0.89	2.52	3.065 (2)	120
N1—H1*B*···O3^ii^	0.89	2.12	2.9378 (19)	153
N1—H1*C*···O3	0.89	2.01	2.900 (2)	179
N1—H1*C*···O2	0.89	2.55	3.158 (3)	126
C8—H8*A*···O1^iii^	0.97	2.45	3.234 (2)	138

Symmetry codes: (i) *x*, *y*+1, *z*; (ii) −*x*+1, −*y*+2, −*z*+1; (iii) *x*, −*y*+3/2, *z*−1/2.
